# Low-Voltage Electrical Stimulation of Beef Carcasses Slows Carcass Chilling Rate and Improves Steak Color

**DOI:** 10.3390/foods10051065

**Published:** 2021-05-12

**Authors:** Christina Bakker, Keith Underwood, Judson Kyle Grubbs, Amanda Blair

**Affiliations:** Department of Animal Science, South Dakota State University, Brookings, SD 57007, USA; Christina.Bakker@sdstate.edu (C.B.); keith.underwood@sdstate.edu (K.U.); amanda.blair@sdstate.edu (A.B.)

**Keywords:** beef, electrical stimulation, glycolytic potential, quality, temperature decline

## Abstract

Electrical stimulation (ES) is used in beef slaughter plants to improve tenderness; however, varying levels of low-voltage ES have not been well characterized. The objective was to evaluate the influence of two levels of low-voltage ES on temperature decline, pH, glycolytic potential, and meat quality. Forty-two beef carcasses were chosen from a commercial packing facility. One side of each carcass received either 40 or 80 volts of ES for 60 s at 45 min postmortem. The paired side of each carcass did not receive ES (Control). Temperature loggers were placed in the sirloin of 12 carcasses to record temperature decline. Longissimus muscle pH was measured at 1, 12, and 24 h, and 3 d postmortem. Strip steaks were fabricated for determination of meat quality. A treatment by time interaction was observed for carcass temperature decline (*p* < 0.001) where ES sides stayed warmer longer than Control sides. A treatment by time interaction was observed for pH decline with Control sides exhibiting an increased pH at 1 h postmortem (*p* < 0.001). Instrumental color values were increased for ES compared to Control sides (*p* < 0.001). These results indicate ES slows carcass temperature decline, hastens initial pH decline, and improves instrumental color. Similar results were observed between the ES treatments indicating either ES level may be used to achieve similar quality characteristics.

## 1. Introduction

Electrical stimulation (ES) is a postmortem intervention utilized to enhance beef quality traits including color, tenderness, and flavor. Electrical stimulation is proposed to improve tenderness by reducing cold shortening [1], disrupting muscle structure [2], and increasing proteolytic activity [3]. Extra low-voltage ES is used on beef carcasses to facilitate the removal of blood from carcasses shortly after exsanguination, while low- and high-voltage ES is used to improve the tenderness and color of beef [4,5,6]. However, there are discrepancies among reports regarding the influence of varying levels of ES on beef quality traits. In a review by Adeyemi and Sazili [7], these discrepancies caused by varying levels of ES on beef quality are highlighted, with some authors reporting positive effects including improvements in tenderness and lean maturity, some reporting negative effects such as reduced color stability and water holding capacity, and others reporting no effect of ES on meat quality, thus concluding the need to further study the effective application of this technology. Throughout the beef industry in the United States, few plants utilize ES in the same manner. Some plants utilize extra low-voltage ES to facilitate blood removal, others apply low- or high-voltage ES to improve tenderness and lean maturity scores, some apply different ES voltage levels throughout the slaughter process, and yet others do not use ES at all. Thus, additional research is necessary to optimize ES applications to ensure beneficial effects are captured and deleterious effects are minimized. Therefore, the objective of this study was to evaluate the influence of two levels of low-voltage electrical stimulation applied at 45 min postmortem on temperature decline, muscle pH, instrumental color, glycolytic potential, and instrumental tenderness. We hypothesized the ES treatments would increase carcass temperature, decrease muscle pH, increase glycolytic potential, improve tenderness, and increase instrumental L* and a* values compared to the non-stimulated sides, with the 80 V ES treatment having a greater impact on these traits than the 40 V treatment.

## 2. Materials and Methods

### 2.1. Carcass Selection and Electrical Stimulation Treatments

Cattle were shipped from feedlots to a commercial slaughter facility and held in lairage following normal plant operating guidelines and United States Department of Agriculture Food Safety Inspection Service regulations for beef slaughter. Source and history of the cattle is unknown. Carcasses (n = 42) were selected for comparison in this study. Three collections were conducted throughout the course of the production day (11 carcasses at 0900 h, 16 carcasses at 1200 h, and 15 carcasses at 1500 h). Carcasses were harvested using standard industry methods. Prior to chilling, paired sides were identified to compare the influence of 2 levels of ES. The left side of the carcasses were subjected to one of two ES treatments, (1) 80 V (ES80; n = 20) and (2) 40 V (ES40; n = 22), 45 min after exsanguination. The right side of each carcass was used as an unstimulated control. For both ES40 and ES80 treatments, the ES was administered through the carcass trolly as it moved over a section of electrically charged rail. Electrical stimulation was applied over a 60 s period where the carcasses received a 4 s pulse of electricity with approximately 2 s between each pulse. The remaining side of each carcass served as a negative control and did not receive ES (Control; n = 42). 

### 2.2. Carcass Temperature and pH

Following application of ES treatments, all carcasses were placed on the same rail in a cooler set to hold at approximately 3 °C for 48 h. Carcass temperature decline was monitored from the timepoint the carcasses entered the cooler on paired sides by inserting a temperature probe (Temprecord Multitrip, Sensitech Inc. Beverly, MA, USA) into the sirloin of both sides of the first 4 carcasses selected at each of the 3 collection time points. Once the cooler was filled with carcasses, the spray chill system was activated to spray water for 1 min every 15 min for 24 h. Upon completion of the 48-h holding period, carcasses were ribbed and allowed to bloom for approximately 30 min before standard carcass data were collected. Longissimus muscle pH was recorded on the medial side of the muscle at the 12th rib position at 1 h postmortem, at the 11th rib at 12 h postmortem, and at the 10th rib at 24 h postmortem to establish a pH decline through the completion of rigor mortis. The pH recordings were taken at different locations on the muscle to avoid influence on pH by utilizing the same probe site. 

### 2.3. Carcass Characteristics and Sample Collection

Fat thickness at the 12th rib (BF) and ribeye area (REA) were measured on both sides of each carcass by South Dakota State University (SDSU) personnel. Fat thickness at the 12th rib and REA measurements of the two sides were averaged and used to calculate USDA Yield Grade. Hot carcass weight (HCW) was recorded from each side and added together for a total hot carcass weight for the carcass. Boneless striploins (IMPS #180) were collected, transported under refrigerated conditions to SDSU, and fabricated into 2.54 cm steaks. Steaks were fabricated in a set order. The first anterior steak was immediately frozen, 3 d postmortem, and used for glycolytic potential (GP) analysis. The second through fourth steaks were aged for 3, 7, or 14 d, respectively, and utilized for Warner–Bratzler shear force (WBSF) and cook loss determination. The second anterior steak was also used to measure ultimate pH, 3 d postmortem. The seventh steak was used to evaluate instrumental lean color for each loin. 

### 2.4. Glycolytic Potential

Glycolytic potential was determined as described by McKeith et al. [8] with minor modifications. Briefly, steaks designated for GP analysis were minced, snap frozen in liquid nitrogen and powdered using a Waring commercial blender (Model 51BL32, Waring Products Division, New Hartford, CT, USA) to produce a homogenous sample. Three g of powdered sample was weighed into a 50 mL plastic conical tube, allowed to thaw, and then homogenized for 75 s in 0.6 N perchloric acid. Samples were then digested using amyloglucosidase and 5.4 N potassium hydroxide and incubated for 3 h, inverting the tubes every 20 min to mix. Upon completion of the incubation step, 3N perchloric acid was added and samples were centrifuged at 4.4 °C for 5 min at 10,000× *g*. Supernatant was collected and stored for analysis. Glucose levels were determined using a glucose assay kit (Glucose (HK) Assay Kit GAHK20, Millipore-Sigma, St. Louis, MO, USA) and absorbance was read at 340 nm (SpectraMax 190, Molecular Devices, San Jose, CA, USA). Lactate levels were determined by adding NAD+ in a glycine buffer to sample aliquots to form NADH. Samples were then read at 340 nm (SpectraMax 190, Molecular Devices, San Jose, CA, USA). Glycolytic potential of each sample was then calculated with the following equation: GP = 2(Glucose absorbance * 111.882) + (Lactate absorbance * 173.22).

### 2.5. Warner–Bratzler Shear Force and Cook Loss

Steaks utilized for WBSF were thawed at approximately 4 °C for 24 h prior to cooking. Steaks were cooked on a clamshell grill (George Foreman Indoor/Outdoor Grill model GGR62, Lake Forest, IL, USA) to an internal peak temperature of 71 °C as indicated by a temperature probe inserted to the geometric center of the steak (Atkins AquaTuff NSF Series Model 351, Middlefield, CT, USA). Steaks were then stored at approximately 4 °C overnight. Four h prior to evaluating shear force values, steaks were placed at room temperature and allowed to equilibrate. Six cores were removed parallel to the direction of the muscle fibers and then sheared once using a Warner–Bratzler shear machine (G-R Electric Manufacturing Company, Manhattan, KS, USA) equipped with a BFG 500 N basic force gauge (Mecmesin Ltd., West Sussex, UK) and peak shear force was recorded for each core. An average shear force value was calculated and recorded for each steak. 

Cook loss was determined on steaks designated for WBSF. Raw steak weight was recorded with a balance (MWP, Cas Corporation, Seoul, South Korea) and after cooking, steaks were allowed to equilibrate to room temperature and weighed again. Cook loss was determined using the following equation: cook loss % = ((raw weight − cooked weight)/raw weight) × 100.

### 2.6. Instrumental Color

Steaks designated for color determination were allowed to bloom for 30 min prior to evaluation. L*, a*, and b* values were recorded at two locations (medial portion of the steak and lateral portion of the steak) using a handheld colorimeter (Chroma Meta CR-410, Konica Minolta, Ramsey, NJ, USA) equipped with a 50 mm aperture, 0° viewing angle, 2° standard observer, pulsed xenon lamp light source, and calibrated with a white tile (L* = 97.38, a* = 0.06, b* = 1.82). Measurements were averaged between both locations for each steak. 

### 2.7. Statistical Analysis

The experiment utilized both sides of 42 carcasses in a completely randomized design. The data analysis was conducted using the MIXED model of SAS software (SAS Institute Inc., Cary, NC, USA) with fixed effect of treatment, random effect of carcass, and Toeplitz covariate structure. Hot carcass weights for both sides of each carcass were added together, and REA and BF measurements were averaged between sides. As HCW from both sides are needed to calculate USDA yield grades, carcass data were analyzed by ES treatment with data reported as ES40 or ES80 treatments. Contrast statements were used to compare Control vs. ES40 and ES80 sides (Control vs. ES), and ES40 vs. ES80 (ES Level). Peak internal cooking temperature was used as a covariate for cook loss and WBSF data. Temperature decline, WBSF, cook loss, and pH were considered repeated measures. Interactions of treatment and time were evaluated where appropriate and are reported when significant. Significance was determined when *p* < 0.05.

## 3. Results

### 3.1. Carcass Characteristics

Carcass characteristics are reported in Table 1. Hot carcass weight did not differ between ES40 and ES80 treatments (*p* = 0.7200). No differences were observed in REA (*p* = 0.6172). Fat thickness measured at the 12th rib was similar between the two treatments (*p* = 0.9482). The lack of differences in HCW, REA, and BF contributed to the absence of differences in overall USDA yield grade (*p* = 0.5000). The absence of differences in carcass characteristics between ES treatments indicates that carcass characteristics likely did not impact carcass chilling or meat quality data.

### 3.2. Carcass Temperature and PH

**Figure 1:**
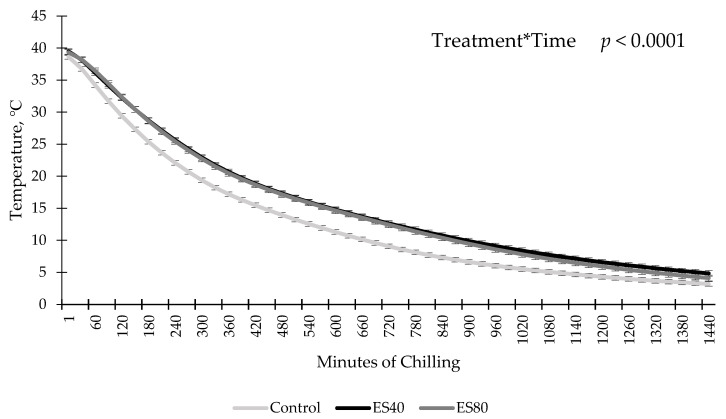
Temperature decline of carcass submitted to low-voltage electrical stimulation (ES) prior to chilling. Data are depicted as least square means ± SEM. Treatments are as follows: Control = no ES, ES40 = 40 V of ES, ES80 = 80 V of ES. Electrical stimulation was applied for 60 s in 4 s on, 2 s off intervals.

An ES by chilling time interaction was observed for temperature decline (*p* < 0.0001; Figure 1). Sides treated with ES prior to chilling had similar temperatures to non stimulated sides at the onset of chilling. By 30 min of chilling, ES sides had increased temperatures compared to sides that did not receive ES, regardless of ES level. This difference persisted until 24 h postmortem when temperature data loggers were removed from the carcasses. No differences in temperature between ES treatments were observed at any time point (*p* > 0.05).

**Figure 2:**
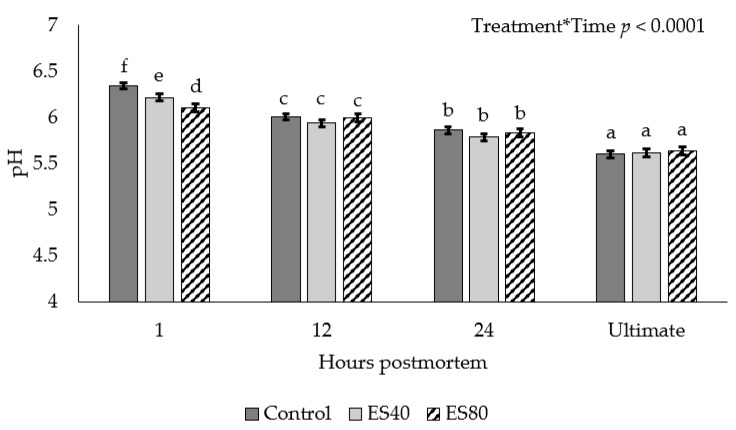
pH decline in beef carcasses subjected to low-voltage electrical stimulation prior to chilling. Data are depicted as least square means ± SEM. Treatments are as follows: Control = no ES, ES40 = 40 V of ES, ES80 = 80 V of ES. Electrical stimulation was applied for 60 s in 4 s on, 2 s off intervals. Measurements were taken at 1, 12, and 24 h postmortem in addition to ultimate pH. ^a–f^ Means with different subscripts differ (*p* < 0.05).

An ES treatment by chilling time interaction was observed for pH decline (*p* < 0.0001; Figure 2). At 1 h postmortem, the ES80 carcasses achieved the lowest pH, ES40 intermediate, and Control sustaining the highest pH value. The pH values measured at 12 and 24 h postmortem, as well as ultimate pH, did not differ among treatments (*p* > 0.05).

### 3.3. Glycolytic Potential

Glucose, lactate, and GP data are reported in Table 2. Glucose concentration did not differ between Control and ES sides (*p* = 0.5825) or between ES treatments (*p* = 0.7308). Additionally, no differences were observed between Control and ES sides (*p* = 0.9557) or between ES levels (*p* = 0.5655) for lactate concentration. Unsurprisingly, based on glucose and lactate results, GP did not differ between Control and ES sides (*p* = 0.6760), or between ES treatments (*p* = 0.5784).

### 3.4. Warner–Bratzler Shear Force and Cook Loss

Steaks from ES sides exhibited decreased shear force values compared to the Control sides (*p* < 0.0220; Table 2). However, when evaluating WBSF data between ES treatments, no differences were observed (*p* = 0.7332). Moreover, an aging day effect was observed for WBSF. Steaks aged 7 d had a greater shear force value compared to steaks aged for 3 or 14 d postmortem (*p* = 0.0021; Table 3).

The percentage of weight lost during cooking did not differ between Control and ES-treated sides (*p* = 0.3753; Table 2) nor were differences observed between sides treated with different ES levels (*p* = 0.8536). An aging day effect was observed for cook loss (*p* = 0.0127; Table 3) with steaks aged 3 d demonstrating less cook loss than steaks aged 7 or 14 d. 

### 3.5. Instrumental Color

Steaks from ES sides were lighter (*p* < 0.0001; Table 4), redder (*p* < 0.0001; Table 4), and more yellow (*p* < 0.0001; Table 4) than control steaks. No differences were observed between ES treatments for lightness (*p* = 0.4582), redness (*p* = 0.9460), or yellowness (*p* = 0.7079). 

## 4. Discussion

Previous research has shown temperature decline trends similar to the current data with ES reported to increase carcass temperature. Bowker et al. [9] measured the temperature decline of the longissimus dorsi in pigs electrically stimulated (six pulses, 60 Hz, 500 V, 1 s on and 2 s off) at 3 min postmortem, and observed an increase in temperature of ES-treated carcasses over the monitoring duration of 56 min. In both cases, the increase in temperature was likely caused by the heat generated by the muscle contractions caused by the ES treatment [9]. Conversely, Wiklund et al. [10] evaluated the temperature decline in the longissimus muscle of red deer carcasses stimulated with 90–95 V of ES for 55 s at the time of exsanguination, and found no differences compared to non-stimulated carcasses. Additionally, Kim et al. [11] evaluated the impact of low-voltage ES (100 V for 30 s) 90 min after exsanguination of beef carcasses, and also observed no differences in the temperature decline of the longissimus dorsi compared to non-stimulated sides. The conflicting results of Wiklund et al. [10] and Kim et al. [11] compared to the current study could be due to differences in species (beef vs. red deer) or time post exsanguination of the stimulation.

Electrical stimulation can cause an increase in the rate of postmortem muscle pH decline by increasing metabolic activity. McKenna et al. [12] observed differences in early pH measurements with ES sides showing decreased pH values compared to non-stimulated sides until 6 h postmortem when pH was similar, until cessation of pH measurements at 24 h postmortem. Moreover, Nichols and Cross [13] noted a similar trend in pH with ES sides displaying a rapid pH decline in the first 6 h postmortem. Kim et al. [11] noted a more dramatic decrease in longissimus muscle pH decline, with non-stimulated sides displaying an increased pH until 24 h postmortem. The rapid pH decline observed was likely caused by the increase in postmortem glycolysis, which resulted in a buildup of lactic acid in muscle at a faster rate than would occur without ES, but resulted in similar ultimate pH values [14,15].

Similar to the current study, Ding et al. [16] observed no differences in glucose or GP values for bison meat from carcasses stimulated with 400 V of ES compared to a non-stimulated control. Conversely, Ding et al. [16] did observe a difference in lactate concentrations; however, the samples were taken from carcasses prior to chilling and rigor mortis. The lack of differences in GP observed in this study was ideal as we could conclude the animals used in this study were at similar metabolic states prior to slaughter. Further, we can conclude that pre-harvest handling did not impact the ability of carcasses in this study to experience a normal rigor processes, such as pH decline, and the differences in pH observed in the present study were likely the result of the ES treatments.

There are several mechanisms by which ES is proposed to improve tenderness [17]. It has been reported that ES disrupts muscle structure at the Z-disk and I-band, causes formation of contraction nodes, and disrupts the integrity of the sarcoplasmic reticulum causing minor separation of myofibrils [2,18,19]. Electrical stimulation has also been proposed to inhibit cold shortening by preventing the temperature of the carcass from declining too rapidly [1,20]. Others hypothesize that improvements in tenderness following ES is caused by the activation of lysosomal enzymes and increased proteolysis while carcass temperature is still increased [2,3,10]. However, some studies have found little or no effects of ES on beef tenderness. Discrepancies between studies could be related to the level of voltage applied, duration of stimulation, or timing of ES after exsanguination [11,21]. However, most studies agree that tenderness development is a complex process that likely involves more than one of the previously discussed mechanisms [2,7,14].

It is unclear why steaks aged for 7 d had increased shear force values compared to steaks aged 3 d, when most normal aging curves would show a decrease in WBSF value as aging day increased during the first few weeks of aging. The steaks utilized for shear force were taken consecutively from the anterior end of the strip loin. Previous research suggests that steaks from those locations should have similar shear force values, likely eliminating the impact of steak location on tenderness [22,23]. The WBSF values for each aging period are below the established threshold for tenderness (4.6 kg) as perceived by consumers as outlined by Shackelford et al. [24]. Additionally, the difference among days is within the 0.5 kg of force described by Miller et al. [25] as the difference in shear force detectable by consumers preparing steak in their own home, indicating that the differences in shear force based on aging day are likely not detectable by the average consumer. 

Cook loss was not impacted by treatment in the current study. These data are similar to the impact of high-voltage ES on cook loss of beef steaks [5,12] or bison steaks [16]. Additionally, Wiklund et al. [10] observed no effect of ES on drip loss of steaks from red deer. However, when evaluating treatment of beef carcasses with 100 V of ES at 1 h postmortem Savell et al. [26] observed increased cook loss for ES vs. control carcasses. However, cook loss in the current study was impacted by aging day. Similar results were observed by Shanks et al. [27] when evaluating cook loss over 35 d postmortem. Increases in cook loss over time may be the result of damage to cellular membranes, which would enable a greater amount of water to leak out of the muscle during cooking [27].

Steaks from ES sides in the current study were lighter, more red, and more yellow than steaks from Control sides. Similar results were observed in beef [5,6,26,28] and in bison [16]. The increased color values can be attributed to the increased oxygen permeability of the meat as a result of the damaged muscle fibers. Weakened muscle structure caused by the intense contractions that occurred during the ES treatment can allow oxygen to penetrate deeper into the muscles, resulting in a thicker layer of oxymyoglobin formation compared to non-stimulated carcasses [6,29,30].

## 5. Conclusions

The goal of this study was to evaluate the effects of varying levels of low-voltage ES on beef quality traits. Collectively, these results demonstrate that low-voltage ES can be an effective means to improve the tenderness and instrumental color scores of beef carcasses without increasing cook loss, potentially improving consumer satisfaction. Within this study the only differences observed between the ES40 and ES80 treatments were the early postmortem pH levels. Thus, beef processing facilities that implement low-voltage ES immediately before carcass chilling may be able to reduce the ES voltage levels to 40 V without detrimentally impacting the meat quality characteristics expected with increased voltages. Additionally, these data show that the desired appearance and palatability benefits of high-voltage ES may be attainable using decreased voltages.

## Figures and Tables

**Table 1:** Least square means for carcass characteristics of carcasses subjected to 40 or 80 V of electrical stimulation for 60 s in 4 s on, 2 s off intervals prior to chilling.

	Treatment ^1^			
Variable	ES40	ES80	SEM ^3^	*p*-Value
Hot carcass weight, kg	427.25	424.30	8.16	0.7200
Ribeye area ^4^, cm^2^	85.06	87.44	4.71	0.6172
12th rib fat thickness ^4^, cm	1.62	1.61	0.14	0.9482
USDA YG ^5^	3.86	3.70	0.23	0.5000

^1^ ES40 = 40 V of electrical stimulation, ES80 = 80 V of electrical stimulation. ^3^ Standard error of means. ^4^ Carcass data measured between the 12th and 13th rib according to USDA standards. ^5^ USDA Yield Grade.

**Table 2:** Warner–Bratzler shear force (WBSF), cook loss, glucose, lactate, and glycolytic potential (GP) of beef carcasses subjected to low-voltage electrical stimulation for 60 s in 4 s on, 2 s off intervals prior to chilling ^1,2.^

	Treatment ^3^			Contrast *p*-Value	
Variable	Control	ES40	ES80	Control vs. ES ^4^	ES Level ^5^
Glucose, µmol/g	0.19 ± 0.006	0.19 ± 0.008	0.18 ± 0.008	0.5825	0.7308
Lactate, µmol/g	0.20 ± 0.005	0.20 ± 0.006	0.19 ± 0.006	0.9557	0.5655
GP, µmol/g	76.10 ± 1.66	76.02 ± 2.18	74.26 ± 2.28	0.6760	0.5784
WBSF, kg	3.84 ± 0.08	3.69 ± 0.10	3.64 ± 0.11	0.0220	0.7332
Cook loss, %	17.94 ± 0.28	18.34 ± 0.38	18.24 ± 0.39	0.3753	0.8536

^1^ Least square means ± standard error of means. ^2^ No interaction was observed for aging day and electrical stimulation treatment (effect of aging day is reported in Table 3). ^3^ Carcasses subjected to 0 (Control), 40 (ES40), or 80 (ES80) V of electrical stimulation. ^4^ Control vs. ES contrast statement compares Control carcasses vs. 40 and 80 V treatments. ^5^ ES Level contrast statement compares 40 vs. 80 V treatments

**Table 3:** Least square means for Warner–Bratzler Shear Force (WBSF) and cook loss values of beef steaks aged 3, 7, or 14 d ^1^ (n = 42/day).

	Days Postmortem ^1^				
Variable	3	7	14	SEM ^2^	*p*-Value
WBSF, kg	3.70 ^a^	3.84 ^b^	3.63 ^a^	0.08	0.0021
Cook loss, %	17.38 ^a^	18.69 ^b^	18.45 ^b^	0.34	0.0127

^1^ No interaction was observed for aging day and electrical stimulation treatment (effect of electrical stimulation treatment is reported in Table 2). ^2^ Standard error of means. ^a,b^ Means with different subscripts indicate a difference within row (*p* < 0.05).

**Table 4:** Instrumental color values of longissimus muscle from beef carcasses subjected to low-voltage electrical stimulation for 60 s in 4 s on, 2 s off intervals prior to chilling ^1.^

	Treatment ^2^			Contrast *p*-Value	
Variable	Control	ES40	ES80	Control vs. ES ^3^	ES Level ^4^
L*	40.38 ± 0.34	42.28 ± 0.46	42.77 ± 0.48	<0.0001	0.4582
a*	24.94 ± 0.30	26.08 ± 0.33	26.06 ± 0.38	<0.0001	0.9460
b*	10.14 ± 0.27	11.30 ± 0.29	11.19 ± 0.34	<0.0001	0.7079

^1^ Least square means ± standard error of means. ^2^ Carcasses subjected to 0 (Control), 40 (ES40), or 80 (ES80) V of electrical stimulation. ^3^ Control vs. ES contrast statement compares Control carcasses vs. 40 and 80 V treatments. ^4^ ES Level contrast statement compares 40 vs. 80 V treatments

## Data Availability

The data presented in this study are available on request from the corresponding author. The data are not publicly available due to privacy restrictions.
